# Reduction of leptin levels during acute exercise is dependent on fasting but not on caloric restriction during chronic exercise: A systematic review and meta-analysis

**DOI:** 10.1371/journal.pone.0288730

**Published:** 2023-11-28

**Authors:** Alexandre Fontana, João Guilherme Vieira, Jeferson Macedo Vianna, Marta Bichowska, Michal Krzysztofik, Michal Wilk, Victor Machado Reis

**Affiliations:** 1 Master in Sports Science, University of Trás-os-Montes e Alto Douro (UTAD), Vila Real, Portugal; 2 Graduate Program in Physical Education, Federal University of Juiz de Fora (UFJF), Juiz de Fora, Brazil; 3 Strength Training Research Laboratory, Federal University of Juiz de Fora (UFJF), Juiz de Fora, Brazil; 4 Faculty of Physical Education, Gdansk University of Physical Education and Sport, Gdansk, Poland; 5 Institute of Sport Sciences, Jerzy Kukuczka Academy of Physical Education in Katowice, Katowice, Poland; 6 Research Center in Sports Sciences, Health Sciences & Human Development (CIDESD), University of Trás-os-Montes e Alto Douro (UTAD), Vila Real, Portugal; General Sir John Kotelawala Defence University Faculty of Medicine, SRI LANKA

## Abstract

**Background:**

The importance of leptin in controlling body mass has recently gained more attention. Its levels are directly associated with the amount of fat mass, but not necessarily dependent on it. Exercise has great potential in reducing leptin levels, however the response of exercise to this cytokine is still not well understood.

**Objective:**

The objective of the review was to analyze the effects of physical exercise on plasma leptin concentration, either acutely (post-exercise/training session) and/or after a training period (short- or long-term), as well as to investigate the existence of possible moderating variables.

**Methods:**

The studies included in this systematic review were published between 2005 and May 2023. Only peer-reviewed studies, available in English, performed with humans that evaluated the effects of any form of exercise on leptin levels were included. The search was conducted on May 03, 2023, in Embase (Elsevier), MEDLINE via PubMed®, and Web of Science (Core collection). The risk of bias in the included trials was assessed by the Physiotherapy Evidence Database tool, considering 11 questions regarding the methodology of each study with 10 questions being scored. The data (*n*, mean, and standard deviation) were extracted from included studies to perform random effects meta-analyses using standardized mean difference between the pre- and post-intervention effects.

**Results:**

Twenty-five studies (acute effect: 262 subjects; short- and long-term effect: 377 subjects) were included in this systematic review and meta-analysis. Short- and long-term physical exercise and caloric restriction plus exercise reduce plasma leptin levels, presenting statistically significant differences (p<0.001); as well as acute effect (p = 0.035), however the latter result was influenced by the pre-exercise meal as shown in the subgroup analysis. In this meta-analysis the effect of moderating factors on leptin reduction, not addressed by past reviews, is verified, such as the relationship with caloric restriction, exercise intensity and pre-exercise meal on acute responses.

**Conclusion:**

Both acute and chronic exercise reduce leptin levels, yet the acute effect is dependent on the pre-exercise meal. In addition to having a long-term reduction in leptin levels, the minimum amount of weekly exercise to have a significant reduction in plasma leptin is 180 minutes of moderate-intensity exercise and 120 minutes of high-intensity exercise.

## Introduction

The concept that adipose tissue is just a fat storage site is outdated. Its influence through the production of cytokines, makes this tissue a powerful endocrine organ, which is able to regulate several other hormones, especially those of hypothalamic origin, such as testosterone, thyroxines and cortisol [1–3]. Leptin, one of the main adipokines, produced mostly in adipocytes, is related to the amount of body fat and continuously signals the central nervous system (CNS) about this amount of stored energy [4]. The level of blood leptin can be reduced, acutely, through prolonged fasting and exercise, otherwise its increase occurs with a pro-inflammatory diet (high-concentration of fat) and/or sedentary lifestyle [4]. Insulin, inflammatory cytokines, glucocorticoids and leptin itself also regulate the expression of leptin through a feedback mechanism [4, 5]. On the other hand, testosterone and catecholamines reduce leptin synthesis [6].

Elevated blood leptin level is associated with obesity and, at the same time, cardiovascular and metabolic complications may be present, such as hypertension, type 2 diabetes mellitus and leptin resistance, which makes hyperleptinemia a general health concern [7, 8]. Leptin has a direct relationship with obesity [4], their levels are comparatively higher than those shown in subjects with a low fat content [9]. Overweight and obese people have difficulty in reducing leptin levels, characterizing a state of resistance to leptin [10–12]. There are several factors that influence and help in leptin resistance, such as failure to transport leptin across the blood-brain barrier, endoplasmic reticulum stress, reduced hypothalamic signaling, dyslipidemia and genetic variations of leptin and its receptors [8, 13]. In addition, leptin has several functions in various physiological systems such as bone metabolism [14–17], immune system [8, 18, 19] and reproduction [20], making leptin a systemic control cytokine.

Obesity can be considered a metabolic complication, which, in addition to the high percentage of fat, is associated with a generalized inflammatory state, which can be observed by the oscillation of some pro-inflammatory cytokines, with leptin being one of the main ones [2]. Despite this, the pulsatile characteristic of leptin is relatively similar in both obese and thin people, but in absolute values the amount of leptin in obese people is significantly higher [1, 21]. It is already clear that trained individuals have lower leptin levels than sedentary individuals, regardless of body mass and fat content, that is, physical exercise has a strong effect on leptin concentration, what is evident when comparing active and inactive individuals, either by body mass index (BMI) or fat content [22–24]. The vast majority of studies involving leptin and physical exercise were conducted using varieties of aerobic exercise, as shown by a recent review [25]. There are studies that observed positive effects of resistance training on body mass reduction [26], and others that show no significant differences [27, 28]. However, such results favoring the reduction of leptin were observed regardless of the change in the percentage of fat, which is often cited and associated as a probable reason for this reduction. However, the reduction of leptin values happens regardless of the type of exercise used [25].

Despite the results of several studies showing positive effects of physical exercise on the reduction of leptin concentration [26, 29], others show contradictory results [28, 30, 31]. Some have observed independent reductions in body fat content [26, 32, 33], while previous reviews associated leptin reduction with reduced fat content [34, 35]. From this perspective, the doubt remains as to what would be the dose of exercise necessary for a reduction in plasma leptin levels to occur, and why in some situations this reduction is not observed. Therefore, the objective of this review was to analyze the effects of physical exercise on plasma leptin concentration, either acutely (post-exercise/training session) and/or after a longer training period (chronic effect), as well as to investigate the existence of possible moderating variables. In addition, the meta-analysis was extended forsome gaps not addressed in previous reviews through subgroup analysis, like pre workout meal, caloric restriction in long-term effects and exercise intensity.

## Materials and methods

A systematic review and meta-analysis of the literature has been performed according to the Preferred Reporting Items for Systematic Reviews and Meta-Analyses (PRISMA) guidelines [36, 37]. The present study did not have a prospectively registered protocol.

### Eligibility criteria

The studies included in this systematic review were published between 2005 and April 2022. As inclusion criteria we adopted: (1) the study should be peer-reviewed, (2) be available in English, (3) performed on humans, (4) use any type of exercise as a form of intervention, (5) evaluate Leptin concentration at rest and soon after exercise and/or after a training period. Furthermore, the exclusion criteria were as follows: (1) be classified as any type of review, (2) include individuals under 18 years of age in the sample, (3) sample composed of individuals over 60 years of age and/or in menopause and/or andropause stage.

Furthermore, only experimental groups were analyzed, control groups were not included in the analysis, since the purpose of the review is to identify the dose response of exercise and not only to identify whether there is a reduction in leptin levels compared to a resting state.

We did not include studies with children or elderly people who were in a phase of low hormonal capacity (menopause and andropause), because there is a strong hormonal contribution to the reduction of leptin levels [38–40], and those studies could impair results of the meta-analysis.

### Information sources

The studies were retrieved from the electronic database search. A search was conducted on May 03, 2023, in Embase (Elsevier), MEDLINE via PubMed®, and Web of Science (Core collection).

### Search strategy

The Cochrane Handbook [41] was used to construct the search strategy. The search strategy consisted of descriptors indexed in Medical Subject Headings (MeSH) combined with Boolean operators (OR and AND). The following descriptors indexed in the MeSH were used: leptin and exercise. The equivalent search syntaxes for all databases were described in the supplementary information.

### Selection of studies

The studies retrieved in each database were processed using Zotero® software (Corporation for Digital Scholarship, Global community) and duplicate studies were manually removed (AF). Initially selecting the studies by title, followed by the selection made by reading the abstracts and finally the full reading of each study classified within the inclusion criteria. Eligibility was assessed independently by two reviewers (AF and VR) and any conflicts were decided by a third reviewer (JGV). The researchers were not blinded to authors, institutions, or journals. When any doubt about the information of the studies arose, an attempt was made to contact the authors by e-mail to clarify.

### Data collection and data items

Two independent reviewers (AF and VR) extracted the data from the full texts. Data were recorded in Microsoft office excel 2013 that were created specifically for this review. The following information was extracted: Sample size, participant characteristics (sex, age, body mass, body fat percentage, body mass index (BMI), weekly exercise frequency, type of exercise, study intervention time, exercise intensity, exercise session time, whether or not there was a pre-exercise meal, whether or not there was caloric restriction and/or diet control during the study and, plasma leptin data before and after intervention). In addition, mean and standard deviation (SD) of the outcomes of interest were extracted. When descriptive data were not reported or were reported graphically in the study, we requested the data from the corresponding author via e-mail. When contact with the corresponding author was not possible, data were extracted from the graphs using ImageJ2 software [42]. In studies that did not present some data, such as the fat content of the sample, a question mark was placed in that category. However, the lack of this information or others were not considered sufficient to remove the studies from this review, because the critical data are those of plasma leptin.

### Risk of bias assessment

The risk of bias assessment of included studies was conducted using the PEDro scale (Physiotherapy Evidence Database) [43], 11 questions referring to the methodology were applied to each study, 10 questions being scored, considering the randomness of the sample distribution, blinding of the sample and evaluators and presentation of statistical results. The higher the score, the higher the quality of the study. All the articles were read in its entirety and subsequently evaluated by two authors (AF and VR.), using the available criteria. The final score higher than 7 was attributed to a study with “high quality”, between 5 and 6 for “moderate quality” and scores lower than 4 were of “low quality”. More information regarding the scale, as well as the content of each question, can be found at: https://pedro.org.au/.

### Effect measures and synthesis methods

The meta-analyses were performed using the Comprehensive Meta-Analysis (CMA) software, version 3.3.070 (Biostat Inc., Englewood, NJ, USA). We performed the meta-analyses, using the standard mean difference (SMD) between the pre- and post-intervention effects of each study as an analysis factor and a confidence interval (CI) of 95%. Significance was stipulated at 5%. Due to methodological variability between studies, the random effects model was used for analysis. The real proportion of the effect size variance was calculated using the I-Squared (I^2^) [44]; the effect size variance was determined by calculating the prediction interval, which determines the dispersion of effects, using specific software for this purpose, also developed by Biostat Inc., which calculates this interval using the average value of the overall effect size, the upper bound of the confidence interval of this effect size, Tau-Squared (T^2^) and the total number of studies to determine this variance interval [45]. From the common moderating variables between the studies (intensity, nutritional support, and intervention time), subgroup analyses were carried out to verify the moderating effect and attempted to determine possible patterns of leptin response through exercise. The analysis were separated between studies that looked at post-exercise/training session effects (soon after until 24 hours) from those that considered short-term exercise effects (1 to 5 weeks) and long-term exercise effects (>5 weeks). For studies in which both situations were observed, data were separated for analysis. Studies which had more than one experimental group, were divided and considered as independent studies, ensuring greater analysis fidelity and case variety. Publication bias was analyzed using Egger’s test and a p-value of 5% was considered significant.

#### Certainty assessment

The certainty of the evidence was assessed using the Grading of Recommendations, Assessment, Development and Evaluation (GRADE) [46] and was performed by two authors (AF and JGV). Initially, GRADE classifies randomized clinical trials as high-quality studies (score 4); the quality of these studies can be lowered according to the identified risk of bias, and can be classified as moderate, low, or very low. The following topics were evaluated: (i) Methodological limitations identified in the studies (risk of bias); (ii) Inconsistency in results (heterogeneity); (iii) Indirect evidence; (iv) Imprecision; (v) Publication bias. For inconsistency, the evidence was downgraded when high and significant heterogeneity was identified in the meta-analysis. The risk of indirect evidence was assessed considering three factors: (1) when interventions differed from the specific intervention desired; and (2) when substitute results were used instead of relevant ones. For imprecision, the evidence was downgraded when a wide CI that could impact outcomes was identified. Regarding publication bias, we deducted one point from the quality score for studies with a significant risk on Egger’s test (p ≤ 0.05).

## Results

### Study selection

**Fig 1:**
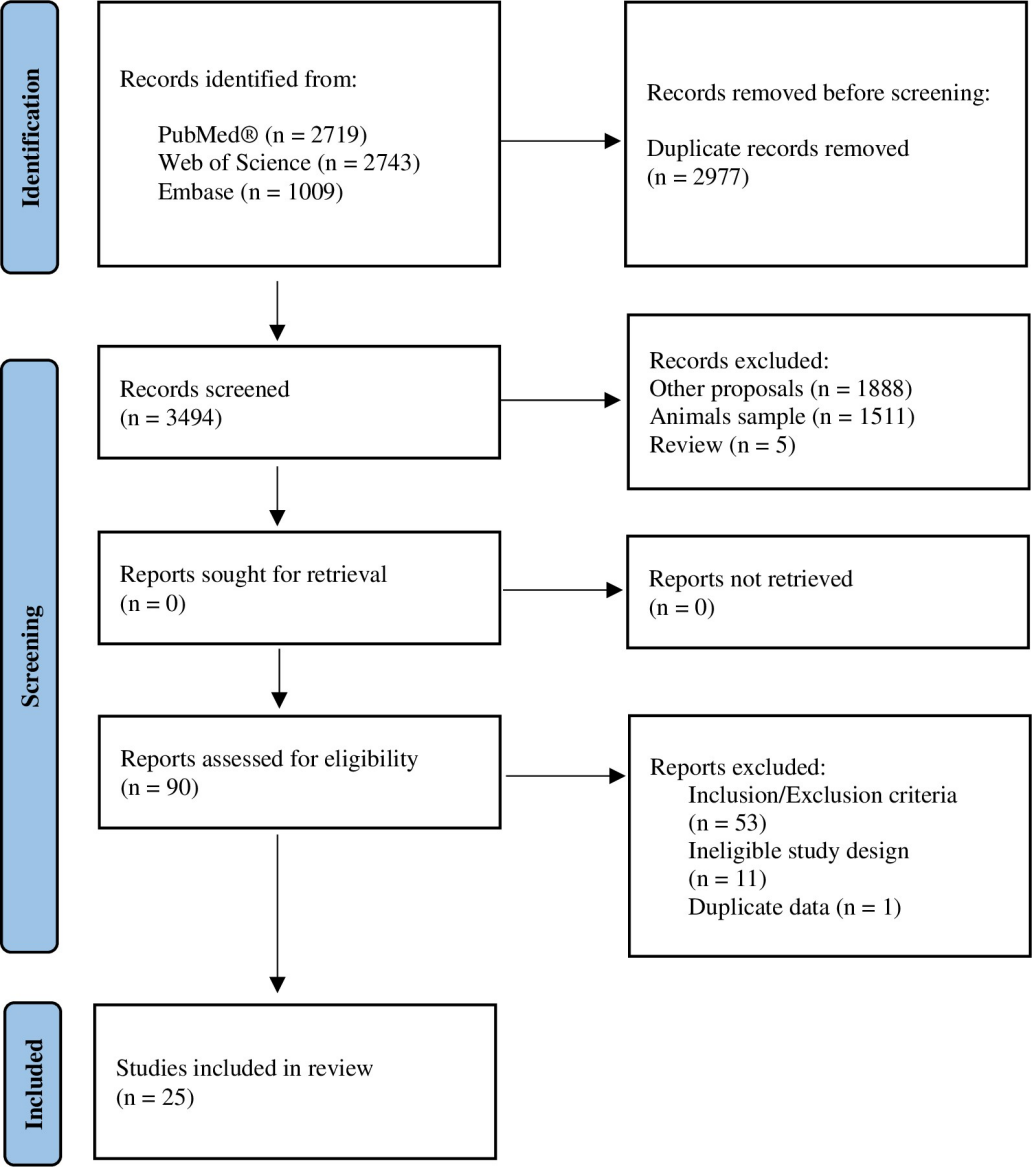
Flow diagram of study selection.

A flow diagram of the literature search is presented in Fig 1. A total of 6471 studies were identified through the databases, 25 studies were selected within the specifications of the inclusion and exclusion criteria. Only one study was removed after the data extraction process [47], as it used the same data that the same author had published previously [48]. We chose to use data from the most recent study.

### Study characteristics

The separation of the studies that evaluated plasma leptin levels after exercise/training session resulted in 23 groups, and for the studies that evaluated leptin levels after a training period, from short- to long-term effect, it resulted in 28 groups. Of all, 7 studies both had leptin analysis data. All included studies were randomized clinical trials. For the effect of exercise after a training period, studies that performed training between one to five weeks were considered short-term, and long-term, studies in which the participants trained for more than 5 weeks (chronic effect). The division of intensity was made by the original classification that each study determined. The training duration time ranged from 30 seconds to 240 minutes in acute effects, and for short- or long-term effect the duration ranged from 90 min/week to 540 min/week. Studies that used a pre-exercise meal were differentiated from those that performed with at least 8 hours of fasting. Studies that had a pre-exercise meal, had it between 1 and 2 hours before. Pre-exercise meals ranged from 75 grams of glucose gel to 870 kcal (~30g protein, ~120g carbohydrate and ~30g fat). The caloric restriction imposed by some studies was performed with at least 20% caloric reduction. Details of the characteristics of the participants and the included studies are presented in Tables 1 and 2.

**Table 1:** Characteristics of the participants.

Study	Participants (*n*)	Gender (M/F)	Age (years±SD)	BMI (kg/m^2^±SD)	FAT (%±SD)	Training status
Joro et al. [32]	17	10/7	26.5 ± 3.2	**Not overtrained:** 22.1 ± 0.5 **Overtrained:** 22.8 ± 0.6	**Not overtrained:** 15.6 ± 2.2 **Overtrained:** 17.2 ± 3.2	Athlete
Yang et al. [33]	18	18/0	29 ± 6	?	14 ± 2	Recreational
Lakhdar et al. [49]	8	8/0	20.7 ±4.8	20.7 ± 1.3	7.8 ± 1.4	Trained
Ahmadizad et al. [27]	32	32/0	23.4 ± 0.6	**Not-Periodized:**27.8 ± 1.7 **Periodized:** 27.5 ± 1.7	**Not-Periodized:** 25.5 ± 2.1 **Periodized:**25.1 ± 2.1	Sedentary
Huuskonen et al. [50]	48	48/0	19 ± 1	**Gr.1**: 27.9 ± 0.5 **Gr.2**: 23.9 ± 0.7 **Gr.3**: 23.6 ± 0.8	**Gr.1**: 22 ± 5 **Gr.2**: 17.8 ± 1.7 **Gr.3**: 15.6 ± 1.8	Trained
Caldeira et al. [51]	20	20/0	26.9 ± 4.7	**HIIT:** 23.31 ±1.43 **SST:** 23.56 ± 5.35	**HIIT:** 18.24 ± 7.7 **SST:** 19.7 ± 5.4	Active
Guerra et al. [52]	15	15/0	23.4 ± 0.6	23.7 ± 5.7	12 ± 7.2	?
Salvadori et al. [53]	16	8/8	38.6 ± 3.9	**Aerobic:** 35 ± 2 **Aerobic + Anaerobic:** 37 ± 2	**Aerobic:** 42.7 ± 5.1 **Aerobic + Anaerobic:** 40.8 ± 5.8	Sedentary
Rosa et al. [48]	10	10/0	27.1 ± 4.8	25.38 ± 0.09	?	Trained
Koehler et al. [54]	6	6/0	25.2 ± 1.0	?	9.6 ± 1.5	Trained
Knuth et al. [55]	26	13/13	43 ± 10	47.6 ± 9.5	49.5 ± 5.6	Sedentary
Zaccaria et al. [56]	7	7/0	27 ± 4.2	22.5 ± 2.03	13.9 ± 3.8	Athlete
Mendham et al. [57]	33	33/0	48.6 ± 6.6	**Cycling:** 29.1 ± 3.8 **Adapted Rugby:** 27.6 ± 2.9	**Cycling:** 28.9 ± 6.3 **Adapted Rugby:** 27.2 ± 2.9	Sedentary
Polak et al. [58]	25	0/25	40.4 ± 6.7	32.2 ± 2.2	38.8 ± 4.2	Sedentary
Bouhlel et al. [59]	9	9/0	19 ± 2	25.1 ± 4.1	16.4 ± 5.5	Athlete
Zoladz et al. [60]	8	8/0	23.0 ± 0.5	22.42 ± 0.49	?	?
Ozcelik et al.[61]	24	0/24	**Exercise:** 43.0 ± 2.1 **Exercise +Meds**: 35.3 ± 3.1	**Exercise:** 39.1 ± 1.9 **Exercise + Meds:** 41.1 ± 2.2	**Exercise:** 47 ± 3.5 **Exercise + Meds:** 45.3 ± 4.3	Sedentary
Jurimae & Jurimae [62]	13	13/0	22.8 ± 4.5	24.1 ± 1.4	13.3 ± 5.8	Trained
Ishigaki et al. [63]	13	13/0	20.5 ± 1.1	19.01 ± 0.75	3.60 ± 0.72	Athlete
Dostalova et al. [64]	25	0/25	**Anorexics**: 22.1 ± 1.0 **Normal:** 21.3 ± 0.9	**Anorexics**: 15.7 ± 0.47 **Normal:** 21.2 ± 0.42	**Anorexics**: 7.1 ± 0.88 **Normal:** 24.3 ± 0.79	Trained
Sari et al. [65]	23	0/23	41.2 ± 10.3	40.7 ± 6.7	?	?
Murakami et al. [66]	42	24/18	51 ± 2.1	27.1 ± 0.3	31.6 ± 0.6	?
Inoue et al. [67]	16	16/0	26 ± 4	?	14.1 ± 3.6	Trained
Olmedillas et al. [68]	16	16/0	35 ± 6.3	?	23.2 ± 5.7	experienced untrained
Oh & Lee [69]	16	0/16	42,5 ± 2,4	?	>30%	Sedentary

*n =* number of participants in each study. M/F = male e female gender. BMI = body mass index. SD = standard deviation.

**Table 2:** Summary and characteristics of the studies included in the review.

Study	Exercise type	Intensity	Intervention time (Weeks)	Leptin before (ng/ml)	Leptin after (ng/ml)
Joro et al. [32]	Cycle ergometer	High	48	**Not-overtrained:** 3.65 ± 0.9 **Overtrained:** 2.15 ± 1.2	**Not-overtrained:** 3.55 ± 0.84 **Overtrained:** 0.75 ± 0.25
Yang et al. [33]	Walk/run	Moderate	0	2.97 ± 0.2	2.1± 0.1
Lakhdar et al. [49]	Cycling	High	24	8.26 ± 4.85	4.55 ± 2.54
Ahmadizad et al. [27]	Resistive	High	8	**Not-Periodized:** 7.3 ± 1.0 **Periodized:** 7.5 ± 1.2	**Not-Periodized:** 7.2 ± 0.9 **Periodized:** 7.3 ± 1.2
Huuskonen et al. [50]	Resistive Walk	Moderate	0	**Gr.1**: 4.9 ± 4.6 **Gr.2**: 10.3 ± 13.4 **Gr.3**: 18.9 ± 14.6	**Gr.1**: 3.2 ± 3.6 **Gr.2**: 4.5 ± 8.5 **Gr.3**: 10.4 ± 10.4
Caldeira et al. [51]	HIIT/SST	High	5	**HIIT:** 3.3 ± 2.7 **SST:** 4.2 ± 2.6	**HIIT:** 2.4 ± 2.7 **SST:** 3.3 ± 1.4
Guerra et al. [52]	Wingate Test	High	0	2.6 ± 4.6	2 ± 3.4
Salvadori et al. [53]	Cycling	High	4	**Aerobic:** 31.6 ± 11.1 **Aerobic + Anaerobic:** 23.1 ± 8.9	**Aerobic:** 10.6 ± 2.4 **Aerobic + Anaerobic:** 17.3 ± 5.6
Rosa et al. [48]	Cycling/Resistive	High	0	**Cycling +Resistive:** 9.97 ± 6.12 **Resistive + Cycling:** 10.07 ± 2.22	**Cycling + Resistive:** 8.37 ± 4.99 **Resistive + Cycling:** 9.21 ± 2.21
Koehler et al. [54]	Cycling	Moderate	1	**Restriction+Exercise:** 1.59 ± 0.28 **Exercise:** 1.40 ± 0.32	**Restriction+Exercise:**0.70 ±0.19 **Exercise:** 1.36 ± 0.26
Knuth et al. [55]	Aerobic/Resistive	High	48	45.2 ± 18.4	3.2 ± 2.4
Zaccaria et al. [56]	Run	High	0	1.12 ± 0.2	0.95 ± 0.2
Mendham et al. [57]	Cycling/Adapted Rugby	High	8	**Cycling:** 11 ± 5.8 **Adapted Rugby:** 9.1 ± 3.8	**Cycling:** 11.6 ± 7.2 **Adapted Rugby:** 6.9 ± 2.3
Polak et al. [58]	Cycling	Moderate	12	24.3 ± 8.7	18.1 ± 8.3
Bouhlel et al. [59]	Cycling/Rugby	High	4	4.50 ± 5.16	3.49 ± 4.27
Zoladz et al. [60]	Cycling	Moderate/ High	0	**Fed:** 2.6 ± 0.95 **Fast:** 2.7 ± 1.2	**Fed:** 2.7 ± 0.95 **Fast:** 3.4 ± 1.5
Ozcelik et al. [61]	Cycling	High	12	**Exercise:** 20.62 ± 1.7 **Exercise + Meds:** 18.92 ± 1.9	**Exercise:** 10.46 ± 1.1 **Exercise + Meds:** 8.12 ± 1.7
Jurimae & Jurimae [62]	Rowing ergometer	High	0	2.7 ± 0.6	2 ± 0.9
Ishigaki et al. [63]	Run	High	1	1.34 ± 0.29	1.49 ± 0.18
Dostalova et al. [64]	Cycle ergometer	Moderate	0	**Anorexics**: 1.5 ± 0.32 **Normal:** 7.4 ± 0.81	**Anorexics**: 1.2 ± 0.6 **Normal:** 6.2 ± 0.3
Sari et al. [65]	Walk	Moderate	4	59.1 ± 20.1	51.2 ±20.5
Murakami et al. [66]	Walk	Moderate	12	7.2 ± 1.6	3.35 ± 0.8
Inoue et al. [67]	HIIT/Resistive	High	8	7.7 ± 4.9	2.9 ± 2.1
Olmedillas et al. [68]	Resistive/Run	High	12	5.5 ± 3.7	3.4 ± 2.5
Oh & Lee [69]	Run/Resistive	Moderate	8	**50% VO**_**2**_ **max:** 13.41 ± 3.06 **80% VO**_**2**_ **max:** 14.84 ± 9.63	**50% VO**_**2**_ **max:** 9.11 ± 6.75 **80% VO**_**2**_ **max:** 9.18 ± 5.48

ng/ml = nanograms per milliliters. HIIT/SST = high intensity interval training/steady-state exercise training. Gr. = group. *Not was specified the studies that measures both effects (acute e long-term). In these cases, only is presented the long-term values for leptin. *When intervention time is zero means that the study only measures the acute effect.

### Risk of bias in studies

The quality analysis and risk of bias conducted using the PEDro scale has an average of 6.4 points (Table 3), resulting in moderate methodological quality and risk of bias of classified studies.

**Table 3:** Risk of bias in the included studies. PEDro scale criteria n° 1 does not contribute to the final score.

Studies	1	2	3	4	5	6	7	8	9	10	11	Score	Quality
Joro et al. [32]	•			•			•	•	•	•	•	6	Moderate
Yang et al. [33]	•	•		•			•	•	•	•	•	7	High
Lakhdar et al. [49]	•	•		•			•	•	•	•	•	7	High
Ahmadizad et al. [27]	•	•		•			•	•	•	•	•	7	High
Huuskonen et al. [50]	•			•			•	•	•	•	•	6	Moderate
Caldeira et al. [51]	•	•		•			•	•	•	•	•	7	High
Guerra et al. [52]	•	•		•			•	•	•	•	•	7	High
Salvadori et al. [53]	•	•		•			•		•	•	•	6	Moderate
Rosa et al. [48]	•	•		•			•	•	•	•	•	7	High
Koehler et al. [54]	•	•					•	•	•	•	•	6	Moderate
Knuth et al. [55]	•						•	•	•	•	•	5	Moderate
Zaccaria et al. [56]	•	•		•			•	•	•	•	•	7	High
Mendham et al. [57]	•	•		•			•	•	•	•	•	7	High
Polak et al. [58]	•	•		•			•	•	•	•	•	7	High
Bouhlel et al. [59]	•						•	•	•	•	•	5	Moderate
Zoladz et al. [60]	•	•		•			•	•	•	•	•	6	Moderate
Ozcelik et al. [61]	•	•		•			•	•	•	•	•	7	High
Jurimae & Jurimae [62]	•	•		•			•	•	•	•	•	7	High
Ishigaki et al. [63]	•			•			•	•	•	•	•	6	Moderate
Dostalova et al. [64]	•			•			•	•	•	•	•	6	Moderate
Sari et al. [65]	•			•			•	•	•	•	•	6	Moderate
Murakami et al. [66]	•	•		•			•	•	•	•	•	7	High
Inoue et al. [67]	•			•			•	•	•	•	•	6	Moderate
Olmedillas et al. [68]	•	•					•	•	•	•	•	6	Moderate
Oh & Lee [69]	•	•		•			•	•	•	•	•	7	High
**Final Average Score**												**6.4**	**Moderate**
**PEDro scale item** **1:** Eligibility criteria specified **2:** Random allocation **3:** Concealed allocation **4:** Group similar at baseline **5:** Subject blinding **6:** Therapist blinding **7:** Assessor blinding **8:** Less than 15% dropouts **9:** Intention-to-treat analysis **10:** Between-group statistical comparisons **11:** Point measures and variability data													

### Main results

#### Acute effect of exercise on leptin levels

The studies that observed the post-exercise/training session effects totaled a sample of 262 participants; of which 16% were female and 84% male; 91% of the total were trained and 9% untrained; 69% were of normal weight (within the range considered by the BMI classification), 18% were overweight and 13% were obese; 54% of the studies had a pre-exercise meal and 46% performed the exercise in fasting.

**Fig 2:**
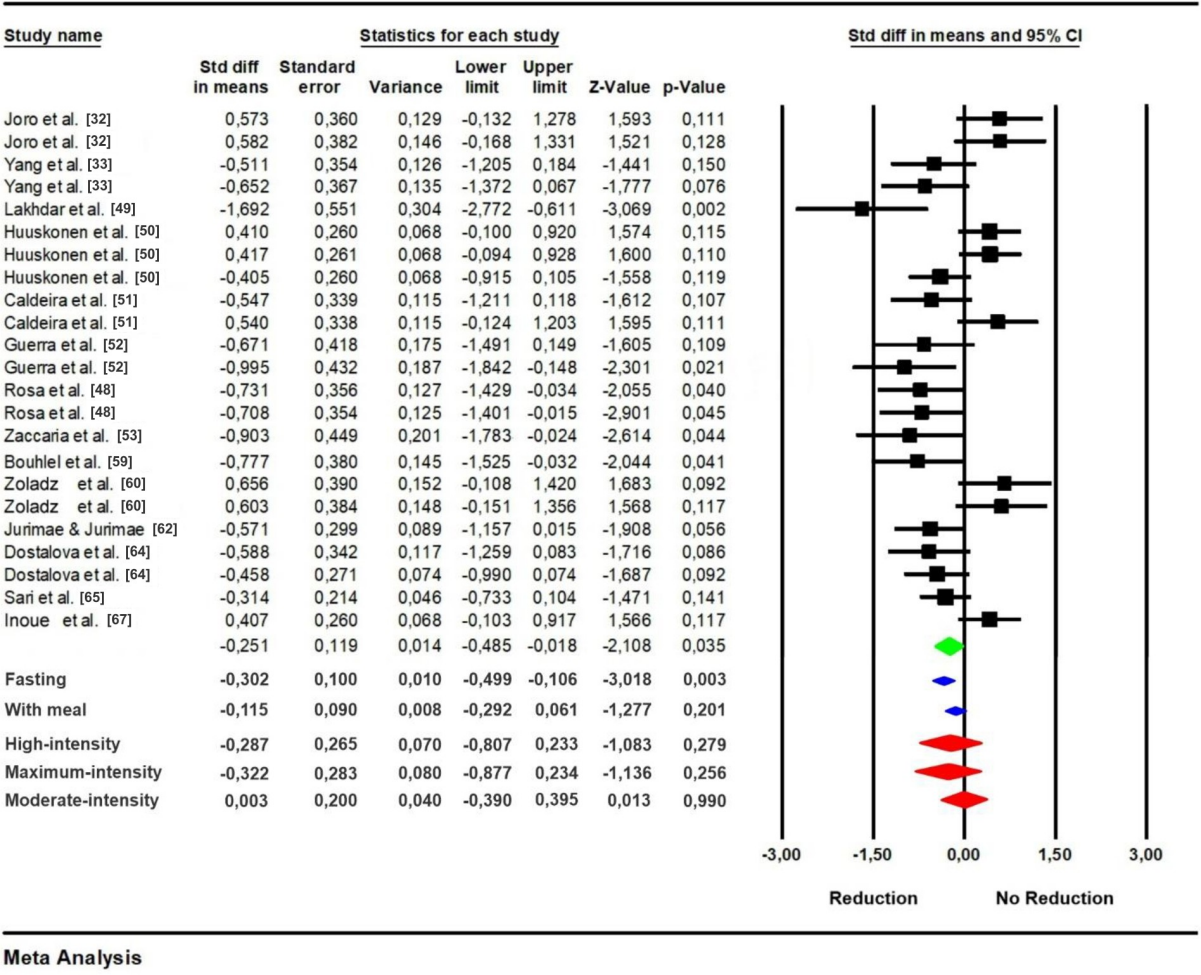
Meta-analysis of the overall effect of studies that observed the effect soon after exercise on plasma leptin levels (green). Subgroup analysis using the use or not of a pre-exercise meal as a moderating variable (blue). Fasting = studies that did not include a pre-exercise meal. With meal = studies that included pre-exercise meal. Subgroup analysis using the exercise intensity as a moderating variable (red).

The general meta-analysis between pre- and post-exercise effect shows that there was a significant reduction in leptin levels, with a value of p = 0035 (Fig 2). The heterogeneity indicated by the I^2^ value (Table 4), confirmed by the p-value, is easily justified by the large methodological and sampling variability of the studies. This does not interfere and has no impact on the final result of the synthesis of this meta-analysis, quite the contrary, it is important to know why the data vary and how much they vary. Using the value of I^2^ to classify heterogeneity as low, moderate or high is considered a mistake by recent analysis [44, 45], because in fact it is not what the value indicates.

**Table 4:** Variance data from post-exercise studies/training session.

Heterogeneity				Tau-Squared			
Q-value	Df (Q)	p-value	I-squared	Tau-Squared	Standard Error	Variance	Tau
69.584	22	0.000	66.947	0.220	0.101	0.010	0.469

Subgroup analysis, intensity of exercise did not influence the acute results of leptin concentration, as no significant differences were observed in any of the strategies (high-intensity: p = 0.279; moderate-intensity; p = 0.990; maximal-intensity: p = 0.256). However, when the use or not of pre-exercise meal was used for subgroup analysis (Fig 2), we noted that studies that did not use this feature had significant reductions p = 0.003, while studies that had a meal prior to exercise did not show the same effect p = 0.201.

**Fig 3:**
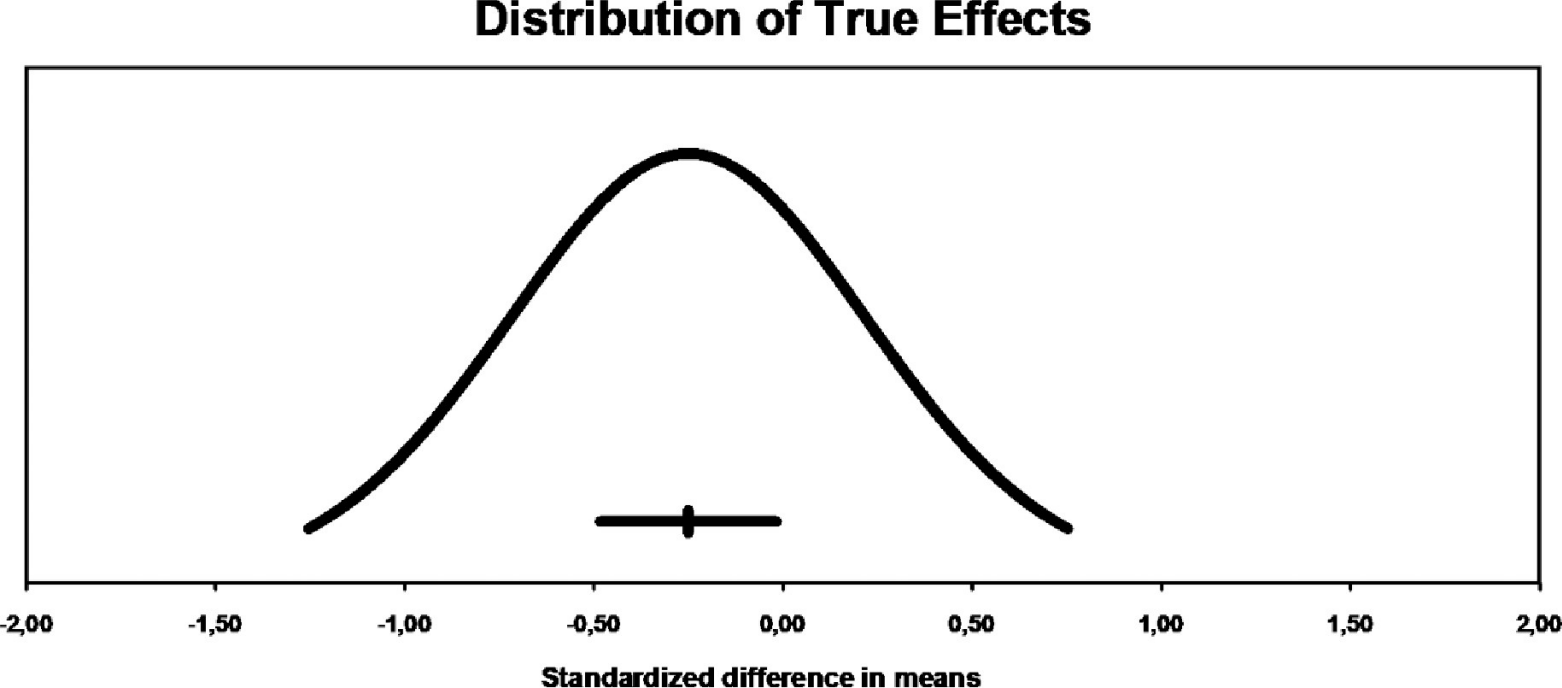
Dispersion of effects in acute effects. The mean effect size is -0.25 with 95% confidence interval located between -0.48 to -0.02. The actual effect size when applied to 95% of the population will be in the range of -1.25 to 0.75.

The prediction range graph (Fig 3), which reveals how much effect sizes vary, presents an average favorable to the reduction of leptin levels, but with a very wide variability, where part of this amplitude is present within the no-effect zone of the exercise (≥ 0), going against the previously mentioned heterogeneity values.

#### Short- to long-term effects of exercise on leptin levels

The meta-analysis of the effect of physical exercise on leptin levels after a short- and long-term totaled a sample of 377 participants; of which 38% were female and 62% male; 37% of the total were trained and 63% were considered untrained; 30% were of normal weight, 42% were overweight and 28% were obese. Of these studies, 80% had no associated caloric restriction and 20% associated caloric restriction with additional physical exercise. In addition, the training period ranged from 1 to 48 weeks, with 65% of the studies having more than five weeks of intervention (long-term) and 35% had less or even 5 weeks of intervention (short-term).

**Fig 4:**
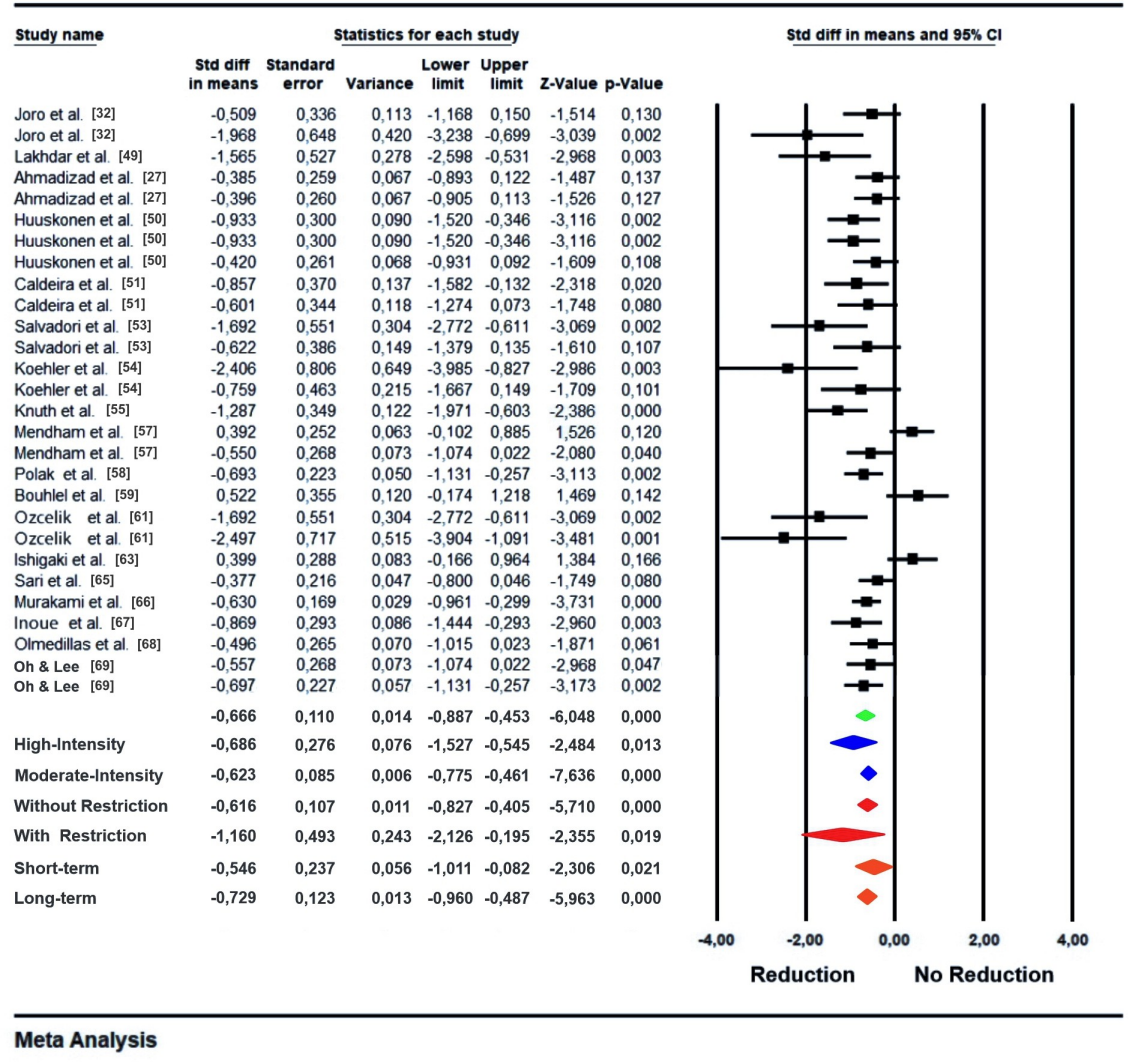
Meta-analysis of the overall effect of exercise on plasma leptin levels after a training period (1 to 48 weeks) (green). Subgroup analysis using exercise intensity as a moderating variable (blue). Subgroup analysis using the presence or absence of caloric restriction in conjunction with exercise (red). With restriction = with caloric restriction diet associated with exercise. With restriction = no caloric restriction diet associated with exercise. Subgroup analysis using the intervention time as moderating variable (orange). Short-term = studies that had less than 5 weeks of intervention. Long-term = Studies that had more than 5 weeks of intervention.

The overall result of the meta-analysis for the short- to long-term effects of exercise on plasma leptin levels (Fig 4) reveals that there is a significant reduction of plasma leptin, with this type of intervention (p < 0.001). Considering variance data from these studies, as well as the acute results, the p-value and I^2^ values confirm the presence of heterogeneity (Table 5). This heterogeneity is justified by the large methodological and sample variability of the studies.

**Table 5:** Study variance data after a training period (short- and long-period).

Heterogeneity				Tau-Squared			
Q-value	Df (Q)	P-value	I-squared	Tau-Squared	Standard Error	Variance	Tau
70.856	27	0.000	67.042	0.191	0.088	0.079	0.436

Subgroup analysis using exercise intensity as the moderating variable (Fig 4) shows that both high-intensity and moderate-intensity training show significant reductions in plasma leptin concentrations after short- and long-term period, p = 0.013 and p < 0.001, respectively. However, it is noteworthy that although the studies have a similar effect (high intensity = -0.686; moderate intensity = -0.623), those that exercised with high-intensity have greater variability (CI = -1.527/-0.545), while studies that used a moderate-intensity of intervention showed a more consistent variability, that is, a smaller amplitude of the confidence interval (CI = -0.775/-0.461). Fig 4 also shows the subgroup analysis separating the studies that used caloric restriction associated with physical exercise from those that did not associate caloric restriction with physical exercise in the observation of changes in leptin levels. With it, it is possible to note that although the results of both strategies are significant (p < 0.001), when there is associated caloric restriction, the effect is almost double (with restriction = -1.160; without restriction = -0.616), but the studies that associated caloric restriction showed a very large variability, as indicated by the confidence interval (CI = -2.126/-0.195). When the intervention time was used as a moderating variable (Fig 4) it is noted that both short- and long-term present significant results regarding the reduction of leptin levels p < 0.001 and p = 0.021, respectively.

**Fig 5:**
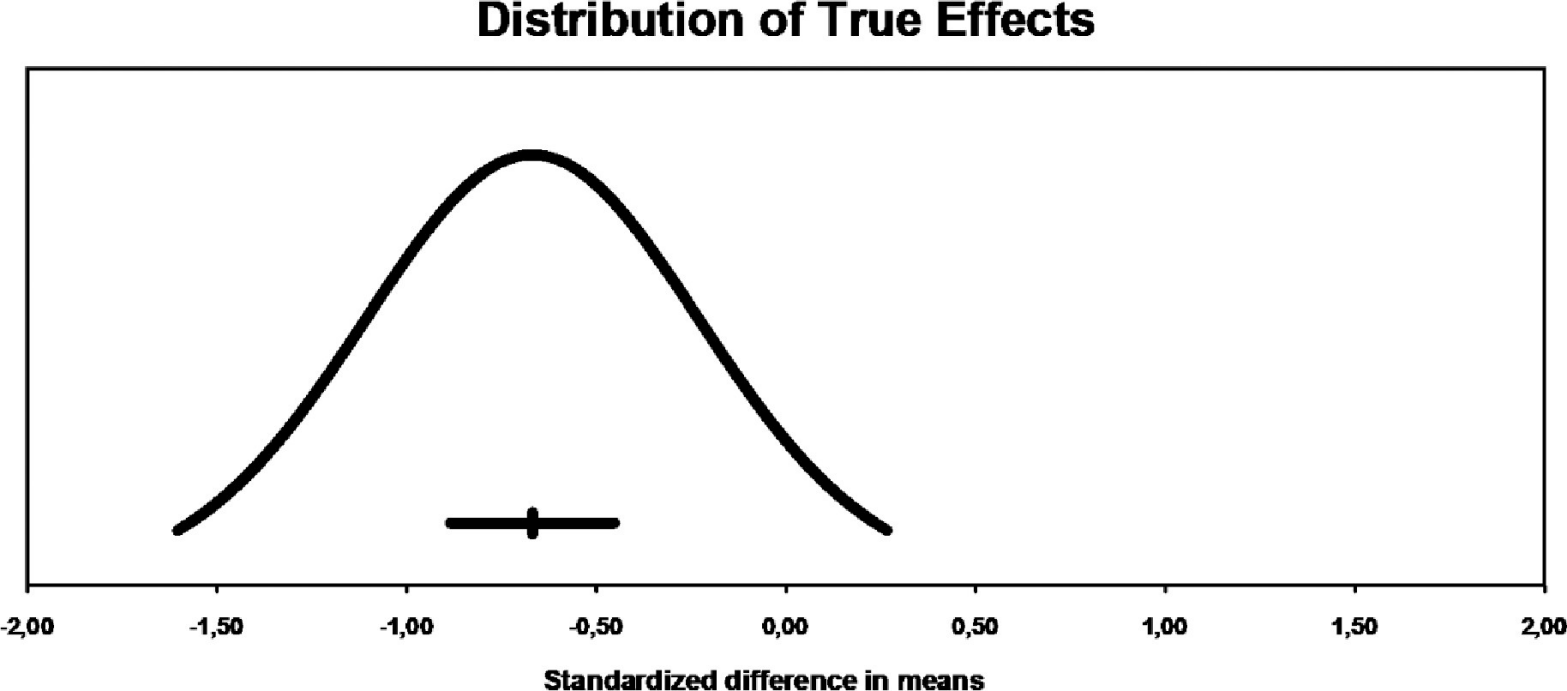
Dispersion of effects in short- and long-term. The mean effect size is -0.68 with a 95% confidence interval located between -0.9 to -0.44. The actual effect size when applied to 95% of the population will be in the range of -1.58 to 0.25.

The real effects distribution graph (Fig 5), which specifies the prediction interval, shows that after a training period of at least moderate intensity, there will be a significant decrease in plasma leptin levels, where the mean confidence interval shows a low variability, but the amplitude of the effect when applied to the population is large. However, this great variability of effect is located, practically in its entirety, in favor of the reduction of leptin values.

#### Certainty assessment

Table 6 shows details of the certainty of evidence (GRADE). There was moderate certainty of evidence for fasting, pre-exercise meal, intensity comparison and caloric restriction (score = 3).

**Table 6:** Certainty of evidence assessment.

Outcomes	Comparison	Summary of findings			Certainty of evidence (GRADE)					
		*k*	*n*	Effect (95% CI)	Risk of bias	Inconsistency	Indirectness	Imprecision	Publication bias	Certainty
Exercise on acute leptin reduction	PRE x POST	23	262	-0.251 (-0.485 to -0.018)	Not serious	Not serious	Not serious	Not serious	Undetected	High
	Fasting x PEM	-	-	-0,199 (-0,330 to -0,068)	Not serious	Not serious	Not serious	Serious	Undetected	Moderate
	MI x HI x MAX	-	-	-0,285 (-0,502 to -0,069)	Not serious	Not serious	Not serious	Serious	Undetected	Moderate
Exercise on short- and long-term leptin reduction	PRE x POST	28	377	-0.666 (-0.887 to -0.453)	Not serious	Not serious	Not serious	Not serious	Undetected	High
	CR x NCR	-	-	-0,642 (-0,845 to -0,435)	Not serious	Not serious	Not serious	Serious	Undetected	Moderate
	MI x HI	-	-	-0,633 (-0,781 to -0,482)	Not serious	Not serious	Not serious	Serious	Undetected	Moderate
	LTE x STE	-	-	-0,694 (-0,902 to -0,473)	Not serious	Not serious	Not serious	Not serious	Undetected	High

PRE = leptin levels before exercise. POST = leptin levels after exercise/training period. PEM = pre-exercise meal. MI = moderate intensity. HI = high-intensity; MAX = maximal-intensity. CR = caloric restriction. NCR = no caloric restriction. LT = long-term effect. STE = short-term effect.— = the same as above.

## Discussion

The objective of this review was to analyze the effects of physical exercise on plasma leptin levels, either acutely (post-exercise/training session) and/or after a training period (short- or long-term), as well as to investigate the existence of possible moderating variables.

Nineteen of the 24 studies used various aerobic exercises as an intervention modality (11 studies used a cycle ergometer), four studies used combined exercise (resistance exercise associated with aerobic exercise) and only one study used only resistance exercise as an intervention method. No analysis was performed on the type of exercise used by the studies, as past reviews showed that there was no difference between the strategies [25], despite having only one study in common with this meta-analysis. Likewise, no comparative analysis of leptin reduction between genders was performed, as it was pointed out in the past that there were no statistically significant differences [70].

### Acute leptin response

To the best of our knowledge, this is the first systematic review involving acute effects of physical exercise on leptin levels, confirming reductions with just one training session. However, in the analysis of the prediction interval, even if the average result is favorable for reduction of leptin levels, when the values are predicted for the population, there may still be situations in which no significant change will be observed, especially if a pre-exercise meal is used. Indeed, we observed that pre-exercise meal has a moderating action on leptin concentration. This implies that the significance shown by the overall result herein should not be considered unilaterally, due to the high amplitude of the prediction interval and its own location. Consequently, it is impossible to conclude that there is indeed an acute significant reduction in leptin levels after exercise/training session, only using the results shown by the general meta-analysis (p = 0.035), as these data suffered a moderating effect of the pre-exercise meal condition. This means that the prediction interval for studies that show the acute effect of physical exercise is also modulated by the moderating variable. Therefore, it can be concluded that reductions in leptin levels occur only when the physical exercise is performed under fasting conditions.

However, some studies that used pre-exercise meal showed significant reductions in plasma leptin [33, 49, 52, 56, 62], representing 50% of the samples that revealed acute significant differences. When isolated, it is observed that intensity of exercise was the determining factor of leptin reduction under these conditions, within a range from high- to maximum-intensity. On the other hand, there were studies in which exercise was performed under fasting conditions and they did not reveal significant reductions in leptin levels [32, 52, 60, 65]. These cases amounted to 35% of all the studies. In this case, when separated and analyzed together, it is possible to point out that the exercise duration was shorter compared to that in studies that achieved significant reductions [48, 59, 64]. However, it is still not possible to identify whether the duration of fasting or the duration of exercise was more significant. It has been suggested that in fasting conditions, the reduction in leptin levels is independent of exercise. This means that even when the duration of exercise performed under fasting is greater, it is not possible to determine when the reduction effect occurs due to the total fasting time or whether exercise accelerates the reduction of leptin levels. Knowing that physical exercise has a direct effect on energy homeostasis, it is logical to state that exercise accelerates the process of reducing leptin levels, though future studies should associate fasting time and the accelerating effect of exercise in reducing leptin levels. Such studies would help to identify more precisely from which moment the decrease in leptin concentration occurs.

It should be noted that the maximum exercise time among the reviewed studies that did not show a reduction of leptin under the fasting condition, did not exceed 45 minutes, while the minimum time following maximum intensity exercise with previous feeding, was 30 seconds. Therefore, under both conditions, with or without fasting, certain requirements are observed for the reduction of leptin levels, dependent on the duration of exercise performed under fasting and dependent on the intensity of exercises performed with a previous meal. Only one study performed maximum intensity fasting exercise [52], and this study observed a reduction in leptin four hours after the cessation of exercise. In this study, a maximal effort test was performed on a cycle ergometer (Wingate test), where one group performed the exercise under fasting and the other ingested 75g of glucose gel 60 minutes before the onset of exercise. The authors commented that the reduction observed in the group that performed the exercise in a fasting condition was associated with an increase in Interleukin-6 (IL-6) and Suppressor of cytokine signaling-3 (SOCS3), which inhibit the signaling cascade for leptin synthesis. The reduction in leptin in the group that consumed glucose before exercise was associated with an increase in insulin, which, added to the effect of exercise, increased the energy imbalance, mainly due to the reduction in blood glucose. However, the authors also observed a significant elevation of IL-6 similar to the fasting group. Thus, it is possible to consider that high-intensity exercise has a greater acute effect on leptin levels, probably because it causes a greater use of plasma glucose, going in favor of the previously mentioned glycostatic model of energy homeostasis control, but also because it increases the production of IL-6. This highlights the importance of the metabolic effect of high-intensity exercise, which is essential for nuclear activation of some specific components involved in leptin control, such as signal transducers and activators of transcription-3 [71].

Importantly, only one study was conducted in an untrained sample [65], while every other was performed in trained subjects. Therefore, all analysis performed in regards to acute effects of exercise on leptin levels are aimed at trained individuals.

### Leptin response to short- and long-term training

As shown above, the results of short- and long-term exercise on leptin indicate a significant reduction in its level. In parallel, the result of the prediction interval when applied to the population herein confirms this result. Contrary to what is shown by the analysis performed on acute effects of physical exercise, practically the entire population can benefit from reducing leptin levels with exercise, as long as it is performed with at least moderate-intensity, even during short intervention periods (≤5 weeks). However, similar to the acute effects of physical exercise, the dispersion of short- and long-term exercise programs is wide, what indicates that several factors influence the magnitude of leptin reduction.

As it turns out exercise intensity was not a factor that influenced post short- or long-term exercise leptin levels. However, as highlighted in the results section, the variability of the confidence interval of the subgroup that performed high-intensity exercise [32, 49, 51, 55, 57, 59, 61, 63, 67] is three times greater than that of the subgroup that trained at moderate-intensity [27, 50, 51, 53, 54, 58, 65, 66, 68]. When analyzed separately, it can be pointed out that the cause for this variability ends up being determined by variables related to the training volume, such as weekly training frequency and duration of each session. By multiplying these two variables and amounting total weekly training time, we have comparable data between these studies, and this data which indicates that the minimum weekly time to observe a significant effect in the reduction of plasma leptin levels is 120 minutes of high-intensity training. As for moderate-intensity training, the minimum weekly time to observe an equivalent effect was 180 minutes. These numbers are higher than the minimum exercise requirements recently proposed by the world health organization [72], which indicate that for moderate-intensity exercises the required time is 150 minutes per week and for high-intensity it is 75 minutes per week.

On the other hand, only two studies that performed high-intensity training did not observe significant differences [59, 63], even performing a training volume higher than the minimum indicated above. These studies were composed of samples of highly trained athletes that underwent significant reductions in the percentage of body fat after the training period. Conversely a study that was also composed of athletes found in one of its samples a significant difference in leptin levels after training [32]. In this case, the sample was composed of athletes in a state of overtraining, which can have a direct influence on the suppression of leptin synthesis, and that according to the authors, although a low level of plasma leptin indicates a better capacity for physical performance, very low levels of this cytokine can indicate a state of overtraining. These athletes showed a 40% lower plasma leptin concentration at the beginning of the training period and an 80% lower level at the end of 48 weeks of training. All this difference was observed for the same level of fat content within the group without a diagnosis of overtraining. Still in that same study, the authors observed a negative relationship between leptin and IL-6 production in the group of athletes with overtraining, which is usually pointed out as an opposite effect, as explained above. Despite this, it seems that plasma leptin values below 2 ng/ml are a strong indication of an overtraining state, though future studies should investigate this relationship in more detail with applicability in identifying this state.

As for the acute effect, this is the first review that includes the effect of exercise training associated with caloric restriction on leptin levels. In this analysis it was pointed out that regardless of the strategy, with or without caloric restriction, a significant difference was identified between pre- and post-training. However, the effect size for the samples that associated caloric restriction was practically double of those that did not use the same strategy, but with a greater variability. Samples submitted to this method had a reduction of at least 50% in plasma leptin levels, even though they had a low percentage of fat. It is interesting to point out that only one study that associated caloric restriction with exercise did not observe significant changes [59], which justifies the variability of the effect size. In this case, the sample consisted of rugby athletes who trained 10 hours a week during the Ramadan period (two meals a day for four weeks), and the authors comment that the caloric restriction imposed during the Ramadan period may have been compensated in the night meal, as an effect of the diurnal leptin drop, which would prevent it from being classified as a restriction, but authors commented that there was a caloric restriction identified through the athletes’ food records. Compared to other studies that associated caloric restriction with exercise and observed reductions in leptin levels, it is noteworthy that most samples were obese and sedentary [55, 61, 66], scenario in which the effect can be doubled, since, as mentioned above, both have a direct impact on plasma leptin levels. The introduction of physical exercise for inactive individuals strongly contribute to the action of sensitivity to leptin at the hypothalamic and peripheral levels, and caloric restriction contributes to the energy suppression caused.

Another study that associated caloric restriction with physical exercise [54], was composed of a trained sample with a low percentage of fat (9.6 ±1.5%), in this study the same sample was submitted to several different strategies, caloric restriction associated with exercise, only caloric restriction and only exercise, with one intervention week for each strategy, in which each intervention was separated by 10 days with no exercise and no caloric restriction. The authors observed that only in the strategies that involved caloric restriction there was a significant reduction in plasma leptin, but it is important to emphasize that the sample was trained, and that the intensity used for the intervention involving exercise was low, relative to the sample’s condition, a factor that may justify the absence of effect when only the exercise was used. One study showed a 90% reduction in leptin levels [55] where the authors compared the effect of caloric restriction associated with exercise in a group that underwent bariatric surgery and another that only used the caloric restriction method. The sample consisted of morbidly obese and sedentary individuals, with initial fat levels close to 50%. The authors observed that both groups had significant reductions in plasma leptin, but the group that only underwent caloric restriction showed a 55% reduction in leptin levels, whereas for the group that associated exercise, the leptin reduction reached an incredible 93%, leaving in average values, from 45.2 ng/ml of initial leptin to 3.2 ng/ml at the end of the intervention interval, presenting in this final phase 30% of fat on average. It is important to point out that the groups had different intervention times, where the group that only had caloric restriction was followed for 48 weeks, while the group that performed exercise with more restriction had 28 weeks of intervention, highlighting the exercise effect. In addition, the group that did not exercise had a significant reduction in lean mass compared to the exercise group. However, it is worth noting that the training consisted of high-intensity sessions, which reached 540 minutes per week.

These results highlight the superiority of exercise in weight-loss strategies and also its importance in leptin suppression compared to strategies that involve only caloric restriction. Thus, it is possible to indicate that caloric restriction associated with exercise causes a reduction of at least 50% in plasma leptin, even in short training periods and regardless of the initial physical state. However, in trained individuals, caloric restriction is most responsible for this reducing effect. For sedentary individuals, the physiological effect of exercise added to the energy suppression of caloric restriction causes a double effect, which underlies the greater effect observed in this systematic review when compared to samples that trained without associated caloric restriction.

Similar to the exercise intensity used, the total weekly exercise time influences the final leptin reducing effect. As discussed, and pointed out before, it takes at least 120 minutes of high-intensity exercise and 180 minutes of moderate intensity exercise for a significant effect on leptin reduction to be observed. Therefore, it is possible to say that the minimum weekly exercise time is intensity-dependent, but that the final reduction effect size is dependent on its total weekly time, that is, the longer the weekly exercise time, the greater the final reduction. Comparatively, to achieve an equivalent final effect, those who use a physical exercise strategy with moderate-intensity must perform training, on average, 50% greater volume than those who use high-intensity exercises. It is important to remember that these values were evident without differentiating strategies with the use of caloric restriction as well as the weekly time, and that they end up influencing only the magnitude of the final reduction effect on leptin levels. However, it is expected that caloric restriction affects energy availability for exercise performance and consequently interferes with the maintenance of training intensity, which is a key factor in plasma leptin reduction. The calorie intake reduction results in a hypothalamic suppression caused by the decrease in leptin levels, causing a lower activity of hormonal precursors, such as thyrotropin-releasing hormone, thyroid stimulating hormone, luteinizing hormone, follicle-stimulating hormone, gonadotropin-releasing hormone and adrenocorticotropic hormone, which act directly in the synthesis of hormones such as testosterone, thyroxines, and cortisol, and these in the use of energy substrates, mainly of fatty origin, which consequently affects the general energy expenditure [3, 73, 74].

Among studies with long-term training, only one [27] did not observe significant leptin reduction after eight weeks of resistance training, which totaled 150 minutes per week of exercises performed at moderate-intensity, on average. On the other hand, the minimum time required to effectively achieve leptin reduction is 180 minutes per week of moderate-intensity training. Thus, the total amount of time per week as a function of intensity did not reach the minimum dose, sufficient to cause a significant reduction in leptin levels, as suggested by this meta-analysis. In addition to this, the 180 minutes weekly benchmark was determined for short training periods. This indicates that a longer training time does not imply leptin reduction, but the weekly time as a function of intensity. Still, it is noteworthy that the experimental samples of the study mentioned above, showed a significant reduction in body fat even without any change in leptin levels, representing a case of reduction independent of the reduction in body fat. Thus, from the point of view of plasma leptin reduction, there seems to be no positive effect when performing a longer training period if the required minimum amount of weekly time is not met, which is dependent on exercise intensity.

So far, the importance of reducing leptin levels has been discussed, regardless of the reduction in body fat, however a marked reduction can be crucial to activate a compensating mechanism for this leptin drop, this compensating mechanism is responsible for inducing an energy replacement, and consequently regaining weight. Studies that looked at weight regain after a high reduction in plasma leptin [75–78] point out that the individuals who had greater reductions in leptin levels were those who returned to their initial weight when they discontinued the intervention strategy. Of these studies, all used the caloric restriction strategy, without any use of associated exercise. In other words, the reduction in leptin levels through caloric restriction causes a rebound effect in the reduction of weight, activating an essential compensating effect in weight regain, modulated by leptin itself. However, it is possible to have significant reductions in plasma leptin, even in short periods of time and without using caloric restriction [51, 53, 65]. These studies showed an average reduction of 30% in leptin levels, while studies involving caloric restriction showed values exceeding 50% [54, 55, 61, 66].

Furthermore, studies that evaluated the effect of exercise on weight maintenance indicate that, unlike caloric restriction, exercise delays weight regain, prolonging the maintenance of reduced weight [79, 80], to which part of the effect acquired through physical exercise is due to appetite suppression. In addition, review studies focusing on the assessment of weight regain, which is directly related to the compensatory effect of leptin drop, indicate that the weekly exercise time is essential in the maintenance or reduction of body mass, with times shorter or up to 150 minutes per week of moderate exercise only significantly reduced the percentage of fat when they associated physical exercise with caloric restriction, as those who had exercise time greater than 150 minutes observed a reduction in the percentage of fat without the involvement of caloric restriction [81–84]. These outcomes are in line with what was proposed by this review and again emphasize the importance of physical exercise in controlling body mass and energy homeostasis.

Finally, among the past review studies [25, 34, 35, 70, 85], those who evaluated acute effects of physical exercise on leptin levels justified the reducing effect by diurnal variation or when there was a large caloric expenditure involved, otherwise no change was noticed. This is partially corroborated by this review, as a moderating effect caused by the pre-exercise meal was identified, which indicates that there is only a reduction in leptin when the exercise is performed in a fasted state, which in turn is in line with the diurnal variation. Furthermore, it was considered by this review that intensity was a decisive factor in the acute reduction of leptin in this interval. For short- to long-term effects, most of the review studies indicated that there was only leptin reduction when associated with it there was a reduction in fat level. This result was also partially reinforced by this review because results were pointed out for both dependent and independent effects of body fat reduction, suggesting a variability as a function of the intervention time. In addition, this review indicates that intensity is a factor that has no influence on the significance of short- or long-term leptin reduction, as long as the minimum weekly exercise time is reached, 180 minutes for moderate-intensity and 120 minutes for high-intensity.

The physical status of the sample was not analyzed in this review, because the vast majority of studies were composed of individuals with normal weight. Nonetheless, it appears that athletes have smaller changes in leptin levels, even with caloric restriction commonly associated with physical exercise. This low responsiveness can result not only from a low-fat percentage, but from high fitness as well. However, athletes diagnosed with overtraining show a marked reduction in leptin levels, despite a fat percentage equivalent to that of athletes outside this condition. Hence, such low responsiveness may be influenced by systemic hormonal imbalance and needs further investigation.

### Limitations

The high heterogeneity found in the analysis was explained in the results section. Furthermore, we believe that the subsequent analysis (subgroups) clarified and identified the factors that in part contributed to this increased value. In the long-term analysis, the number of studies that performed caloric restriction and exercise were low compared to those that performed exercise alone. This contributed to the higher inconsistency.

All results should consider the biases found by the classification of the study quality scale and the certainty of evidence.

## Conclusions

A strong dependence on exercise intensity is associated with an acute reduction in leptin when there is a pre-exercise meal; but in conditions where exercise is performed under fasting, the reducing effect depends on the volume of exercise, which can accelerate the process of diurnal leptin decay. Intensity dependence also is evident for effects in short- or long-term training. The minimum weekly time required to effectively reduce plasma leptin levels is 120 minutes of high-intensity exercise or 180 minutes of moderate-intensity exercise per week.

The marked reduction in leptin generates an energy compensating effect, but this effect is more evident when there is caloric restriction involved, because when exercise is associated with caloric restriction, this compensating effect is of lesser magnitude. This topic needs further investigation.

Furthermore, future studies should focus on more accurate identification of the difference between fat reduction effect caused by fasting and exercise. Furthermore, an approach focused on molecular crosstalk mechanisms will help in the interpretation of the systemic effect of leptin, especially during exercise. Still, future studies should clarify the effect of caloric restriction and physical exercise on appetite control and its relationship with changes in leptin level.

## Supporting information

S1 Checklist PRISMA checklist. (DOC)

S1 Text Search strategy syntax web of science. (DOCX)

S2 Text Search strategy syntax PUBMED. (DOCX)

S3 Text Search strategy syntax EMBASE. (DOCX)
