# Supplementary material for: Reduction of leptin levels during acute exercise is dependent on fasting but not on caloric restriction during chronic exercise: A systematic review and meta-analysis
Source: PLoS One. 2023 Nov 28;18(11):e0288730. doi: 10.1371/journal.pone.0288730 (PMC10684016; doi:10.1371/journal.pone.0288730)
Supplement: S1 Text — (DOCX) [file pone.0288730.s002.docx]

**Search Strategy Syntax**

**Web of Science:**

“Leptin” (Tópico) AND “Exercise” (Tópico)

2,743 results

May 03, 2023
