# Supplementary material for: Reduction of leptin levels during acute exercise is dependent on fasting but not on caloric restriction during chronic exercise: A systematic review and meta-analysis
Source: PLoS One. 2023 Nov 28;18(11):e0288730. doi: 10.1371/journal.pone.0288730 (PMC10684016; doi:10.1371/journal.pone.0288730)
Supplement: S2 Text — (DOCX) [file pone.0288730.s003.docx]

**Search Strategy Syntax**

**PubMed:**

#1 = (“Leptin”[MeSH Terms] OR “Leptin”[Title/Abstract] OR “Obese Protein”[Title/Abstract] OR “Obese Gene Product”[Title/Abstract] OR “Gene Product, Obese”[Title/Abstract] OR “Ob Gene Product”[Title/Abstract] OR “Gene Product, Ob”[Title/Abstract] OR “Ob Protein”[Title/Abstract])

#2 = (“Exercise”[MeSH Terms] OR “Exercise”[Title/Abstract] OR “Exercises”[Title/Abstract] OR “Physical Activity”[Title/Abstract] OR “Activities, Physical”[Title/Abstract] OR “Activity, Physical”[Title/Abstract] OR “Physical Activities”[Title/Abstract] OR “Exercise, Physical”[Title/Abstract] OR “Exercises, Physical”[Title/Abstract] OR “Physical Exercise”[Title/Abstract] OR “Physical Exercises”[Title/Abstract] OR “Acute Exercise”[Title/Abstract] OR “Acute Exercises”[Title/Abstract] OR “Exercise, Acute”[Title/Abstract] OR “Exercises, Acute”[Title/Abstract] OR “Exercise, Isometric”[Title/Abstract] OR “Exercises, Isometric”[Title/Abstract] OR “Isometric Exercises”[Title/Abstract] OR “Isometric Exercise”[Title/Abstract] OR “Exercise, Aerobic”[Title/Abstract] OR “Aerobic Exercise”[Title/Abstract] OR “Aerobic Exercises”[Title/Abstract] OR “Exercises, Aerobic”[Title/Abstract] OR “Exercise Training”[Title/Abstract] OR “Exercise Trainings”[Title/Abstract] OR “Training, Exercise”[Title/Abstract] OR “Trainings, Exercise”[Title/Abstract])

#1 AND #2 = ((("Leptin"[MeSH Terms] OR "Leptin"[Title/Abstract] OR "Obese Protein"[Title/Abstract] OR "Obese Gene Product"[Title/Abstract] OR "Gene Product, Obese"[Title/Abstract] OR "Ob Gene Product"[Title/Abstract] OR "Gene Product, Ob"[Title/Abstract] OR "Ob Protein"[Title/Abstract]))) AND ((("Exercise"[MeSH Terms] OR "Exercise"[Title/Abstract] OR "Exercises"[Title/Abstract] OR "Physical Activity"[Title/Abstract] OR "Activities, Physical"[Title/Abstract] OR "Activity, Physical"[Title/Abstract] OR "Physical Activities"[Title/Abstract] OR "Exercise, Physical"[Title/Abstract] OR "Exercises, Physical"[Title/Abstract] OR "Physical Exercise"[Title/Abstract] OR "Physical Exercises"[Title/Abstract] OR "Acute Exercise"[Title/Abstract] OR "Acute Exercises"[Title/Abstract] OR "Exercise, Acute"[Title/Abstract] OR "Exercises, Acute"[Title/Abstract] OR "Exercise, Isometric"[Title/Abstract] OR "Exercises, Isometric"[Title/Abstract] OR "Isometric Exercises"[Title/Abstract] OR "Isometric Exercise"[Title/Abstract] OR "Exercise, Aerobic"[Title/Abstract] OR "Aerobic Exercise"[Title/Abstract] OR "Aerobic Exercises"[Title/Abstract] OR "Exercises, Aerobic"[Title/Abstract] OR "Exercise Training"[Title/Abstract] OR "Exercise Trainings"[Title/Abstract] OR "Training, Exercise"[Title/Abstract] OR "Trainings, Exercise"[Title/Abstract])))

Quoted phrases not found: Gene Product, Obese, Gene Product, Ob, Trainings, Exercise

2,719 results

May 03, 2023
