# Supplementary material for: Reduction of leptin levels during acute exercise is dependent on fasting but not on caloric restriction during chronic exercise: A systematic review and meta-analysis
Source: PLoS One. 2023 Nov 28;18(11):e0288730. doi: 10.1371/journal.pone.0288730 (PMC10684016; doi:10.1371/journal.pone.0288730)
Supplement: S3 Text — (DOCX) [file pone.0288730.s004.docx]

**Search Strategy Syntax**

**Embase:**

#1 = 'leptin'/exp

#2 = 'exercise'/exp

#1 AND #2

#3 AND [embase]/lim NOT ([embase]/lim AND [medline]/lim)

1,009 results

May 03, 2023
